# Mechanochemical Near‐Ambient Synthesis of C_2_N Materials From HAT‐CN and its Precursors

**DOI:** 10.1002/cssc.70678

**Published:** 2026-05-10

**Authors:** Pascal Dippner, Sven Grätz, Jonas Lins, Torsten Gutmann, Lars Borchardt

**Affiliations:** ^1^ Inorganic chemistry I Ruhr‐Universität Bochum Bochum Germany; ^2^ Eduard‐Zintl‐Institute for Inorganic and Physical Chemistry Technische Universität Darmstadt Darmstadt Germany; ^3^ Department of Chemistry, Physical Chemistry Universität Paderborn Paderborn Germany

**Keywords:** carbon, energy conversion

## Abstract

C_2_N‐type carbon materials are typically obtained through high‐temperature treatment of nitrogen‐rich molecular precursors under inert atmosphere. Herein, we demonstrate mechanochemical approaches that enable the synthesis of C_2_N materials, namely by (i) the conversion of hexaazatriphenylenehexacarbonitrile (HAT‐CN) and by (ii) a one‐pot route starting from its molecular precursors, hexaketocyclohexane, and diaminomaleonitrile. Compared with conventional pyrolytic methods, mechanochemical approaches afford higher yields while significantly reducing energy input, thereby improving overall sustainability. The results highlight the decisive role of mechanical energy in directing carbon–nitrogen framework formation and demonstrate mechanochemistry as a versatile alternative to thermal routes for C_2_N synthesis.

## Graphical abstract

The synthesis of C2N‐type materials was dominated by temperature‐based materials. Herein, we present two innovative mechanochemical pathways to synthesize those materials, revealing the significant impact of energy input per single‐impact on the structure and yield.

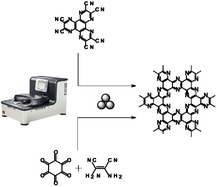

## 1 Introduction

Functional carbon materials play a central role in gas sorption and electrochemical energy storage, providing sustainable and low‐cost solutions for applications such as supercapacitors, batteries, and fuel cells [1, 2, 3, 4, 5, 6, 7, 8, 9].

Their limited interaction with polar media, including water, originates from the hydrophobic π‐conjugated electronic structure [9, 10]. Introducing heteroatoms—primarily nitrogen, but also sulfur, phosphorus, or boron—modifies the framework and surface polarity, thereby enhancing hydrophilicity and chemical reactivity [11, 12, 13, 14].

Among these dopants, nitrogen has attracted particular attention. Nitrogen‐doped carbons function as efficient electrocatalysts for the oxygen reduction reaction in fuel cells [15, 16, 17], serve as effective hosts for Li‐polysulfides in lithium–sulfur batteries [15, 16] and act as selective CO_2_ adsorbents for carbon capture applications [18].

To introduce nitrogen into preformed carbon frameworks, several post‐functionalization strategies have been developed, including mechanochemical approaches [19], plasma‐based treatments [20], and high‐temperature gas‐phase methods employing reactive nitrogen species [19, 20, 21]. In parallel, bottom‐up syntheses via pyrolysis of nitrogen‐rich precursors—such as melamine‐based polymers [22], dicyandiamide [23] or polyacrylonitriles [24]—enable higher nitrogen incorporation. A particularly promising precursor is hexaazatriphenylene (HAT‐CN), an intrinsically nitrogen‐rich molecule that yields carbons with exceptionally high nitrogen contents. Oschatz and coworkers demonstrated its thermal conversion into a C_2_N‐type framework containing >30 wt% nitrogen [25]. In their approach, HAT‐CN is obtained by refluxing hexaketocyclohexane and diaminomaleonitrile in acetic acid, followed by treatment in nitric acid, and subsequently pyrolyzed at 550°C under inert atmosphere to give a highly hydrophilic, zeolite‐like C_2_N‐type material [9, 25, 26].

In this work, we present a novel near‐ambient synthesis route for C_2_N‐type materials, based on mechanochemistry.

Mechanochemistry drives chemical transformations using mechanical energy provided by ball milling, thereby avoiding solvent usage and thermal treatment [16, 27, 28, 29, 30].

Its solid‐state nature allows unique reaction pathways inaccessible by conventional wet‐chemical methods [31].

While various nitrogen‐containing carbons have been prepared mechanochemically—for example, from lignin, urea, and K_2_CO_3_, yielding up to 6 wt% nitrogen—these approaches still require a subsequent high‐temperature pyrolysis step (typically 800°C) [16, 30]. Casco et al. reported the purely mechanochemical formation of carbon materials at room temperature by milling hexachlorobenzene [32] or cyanuric chloride [31] with calcium carbide; however, the highly reactive carbide demanded inert milling conditions.

**FIGURE 1:**
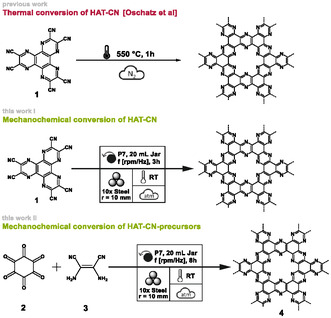
Classical thermal conversion [25] of HAT‐CN (1) and the herein presented mechanochemical conversion of HAT‐CN (1), respectively hexaketocyclohexane (2) and diaminomaleonitrile (3) into C_2_N materials (4).

Building on these insights, the present work introduces two mechanochemical routes to C_2_N‐type materials with nitrogen contents exceeding 30 wt%, achieved without additional heating or inert atmosphere (see Figure 1): (i) the direct conversion of commercially available HAT‐CN into a C_2_N‐type material and (ii) a one‐pot synthesis starting from the HAT‐CN precursors hexaketocyclohexane (HKCH) and diaminomaleonitrile (DAMN).

## 2 Results and Discussion

Three types of mills were employed—a MM500 mixer mill, a P7 planetary mill, and a high‐energy Emax ball mill—each providing distinct milling dynamics and energy impacts (see Figure S1). Milling parameters such as vessel and ball material (PP, Si_3_N_4_, ZrO_2_, steel, WC), time (60–480 min), and frequency (400–2000 rpm, fixed at 35 Hz for the MM500) were optimized.

The resulting materials were evaluated, primarily by yield, purity, and nitrogen content. Additional analysis included the investigation of surface characteristics.

### 2.1 Synthesis of the C_2_N‐Material from HAT‐CN

In a typical synthesis, 600 mg of HAT‐CN is milled with ten steel balls (*Ø* = 10 mm) in a P7 planetary ball mill at 800 rpm for 180 min, using a steel vessel (*V* = 20 mL). The resulting black powder was worked up by washing it with 50 mL of hydrochloric acid and 300 mL of water and dried at 80°C overnight.

**FIGURE 2:**
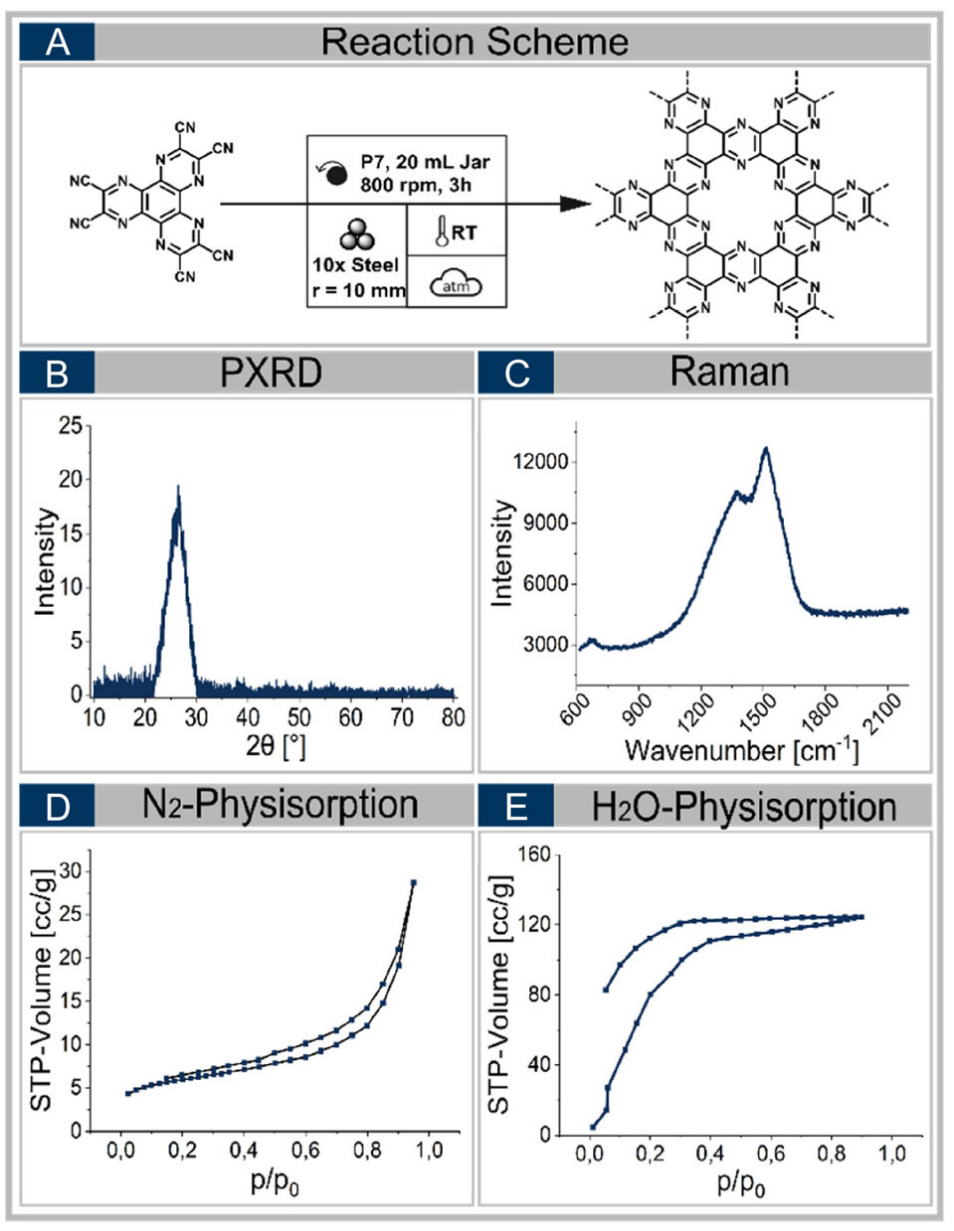
Analysis results of the HAT‐CN conversion **I‐13** in the P7 ball mill, using steel as vessel and ball material (A). It contains the X‐ray diffractogram (B), the Raman spectrum (C), the nitrogen physisorption isotherm (D) and the water physisorption isotherm (E).

Its analysis by powder X‐ray diffraction (see Figure 2B) reveals a broad signal at 2*θ* = 27°, characteristic of amorphous carbon, and no crystalline byproducts are detected in the optimized samples [33, 34]. Raman spectra consistently show the presence of D‐ and G‐bands, verifying the formation of sp^2^‐hybridized carbon domains (see Figure 2C) [35, 36, 37, 38]. A blue shift of the D‐band and a pronounced red shift of the G‐band indicate the electronic influence of nitrogen dopants on the carbon framework, consistent with previous reports for N‐doped carbon materials [35, 36, 37, 38]. The specific surface area (SSA) remains constant at ˜20 m^3^/g, almost unaffected by the variation of mechanochemical parameters and ball mills. All obtained isotherms are type II, indicatingthat the material is essentially nonporous (see Figure S4) [39]. The water adsorption isotherm reveals a high chemical affinity to water, as the material—even with a low SSA—takes up water at low vapor pressure, corresponding to the high initial slope of the isotherm (see Figure 2E). The maximum water uptake is 5.6 mmol g^−1^ at saturation humidity of p/p_0_ = 0.90.

The elemental analysis confirms high nitrogen contents (>30 wt%) across all samples, demonstrating that the mechanochemical synthesis efficiently preserves nitrogen from the HAT‐CN. Minor oxygen contributions (6–10 wt%) originate from processing under ambient atmosphere. Together, these results indicate that the mechanochemical route yields a nitrogen‐rich carbon phase of C_2_N‐type. Some of the elemental analyses indicate that small amounts of iron or zirconium abrasion arise from high‐energy milling. Iron contamination from steel media is effectively removed during the acidic washing step, and no residual metal is detected in the final C_2_N materials. Traces of chlorine are detected as remaining from the acidic treatment by XPS and SEM/EDX.

XPS measurements reveal multiple carbon, nitrogen, and oxygen chemical environments characteristic of nitrogen‐enriched carbon materials. The C 1s spectrum is primarily composed of C–O and polar C = N species, accompanied by contributions from aromatic/graphitic C–C, aliphatic C–C/C–H, and carbonyl functionalities such as amide or imide groups. The N 1s region indicates the presence of pyridinic and iminic nitrogen, amine/amide nitrogen, as well as protonated or quaternary nitrogen species. Signals in the O 1s spectrum are attributed to carbonyl or N–O oxygen and C–O functionalities, with partial contributions arising from adsorbed water (see Figure S2).

**FIGURE 3:**
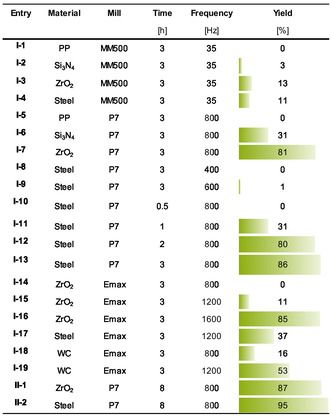
Yields and parameters for synthesis experiments. The vessel and ball materials were varied between polypropylene (PP), silicon nitride (Si_3_N_4_), zirconium dioxide (ZrO_2_), steel, and tungsten carbide (WC).

Screenings of milling parameters time, frequency, and ball mass reveal that the conversion depends on applied mechanical energy. Even slight reductions in milling time or frequency result in markedly lower yields. Consistently, heavier milling materials—with higher impact forces—produce higher conversions (see Figure 3). Likewise, the milling device strongly influences the outcome—yields rise from the MM500 mixer mill to the P7 planetary mill and reach their maximum in the high‐energy Emax ball mill. Almost no product was obtained after only 30 min of milling with steel balls. Samples milled in the MM500, especially with polypropylene (PP) balls, remained brownish rather than black, indicating incomplete conversion (see Figure S4). On the other hand, under excessively harsh conditions, however, pronounced material abrasion occurs, particularly with tungsten carbide (WC) components leading to artificially high apparent yields. These observations confirm that a critical mechanical energy threshold must be exceeded to induce the transformation of HAT‐CN into the C_2_N‐type material. The energy transfer efficiency varies between mills due to differences in vessel geometry and ball trajectories, explaining yield discrepancies under otherwise comparable conditions [40].

**FIGURE 4:**
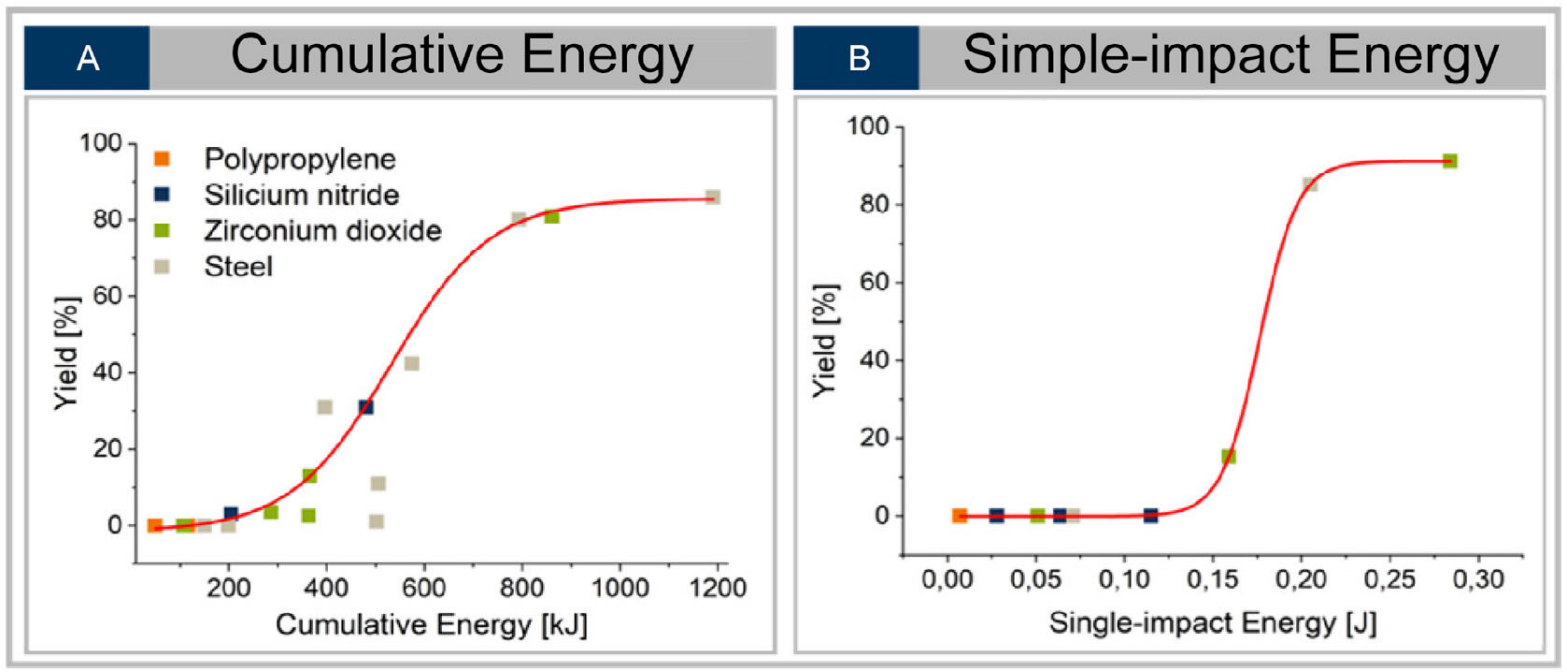
Yields of products of reaction series **I** as function of cumulative energy input (A) and of reaction series **V** as function of single impact energy input (B), calculated, with the Lungerich tool [41].

The correlation between energy input, calculated with the Lungerich tool [41] and product yield reveals that the cumulative mechanical energy correlates positively with the reaction yield (see Figure 4A). However, when the impact energy per collision is reduced—for instance, by decreasing the milling frequency—the yield decreases. This was investigated further by a series of experiments (**V**), where the cumulative energy was kept constant at *E*_cum_ = 800 kJ, while the single‐impact energy was varied by different milling materials and frequencies for each experiment (see Figure 4B).

The results indicate that not only the overall energy dose but also the distribution of energy among individual impacts plays a decisive role in determining the reaction efficiency. Lower impact energies fail to overcome local activation barriers, resulting in less efficient bond activation and fewer effective reaction events. A nonlinear regression of the data on yield and single‐impact energy results in an onset value of 0.15 J, which is interpreted as the critical threshold energy that must be achieved by one impact to overcome the local activation barriers and lead to a successful reaction.

### 2.2 Two‐Step One‐Pot Synthesis of the C_2_N‐Material

In the next step, we directly synthesized C_2_N materials from hexaketocyclohexane and diaminomaleonitrile mechanochemically, thus eliminating the need to synthesize [42], isolate, or purchase the intermediate product, HAT‐CN. In short, hexaketocyclohexane and diaminomaleonitrile were milled together in the P7 planetary ball mill for 8 h at 800 rpm and subsequently washed with water and hydrochloric acid.

The transformation was successfully achieved with an excellent yield of 94.6%. After workup, the resulting black product powder was characterized the same way as the HAT‐CN converted material.

**FIGURE 5:**
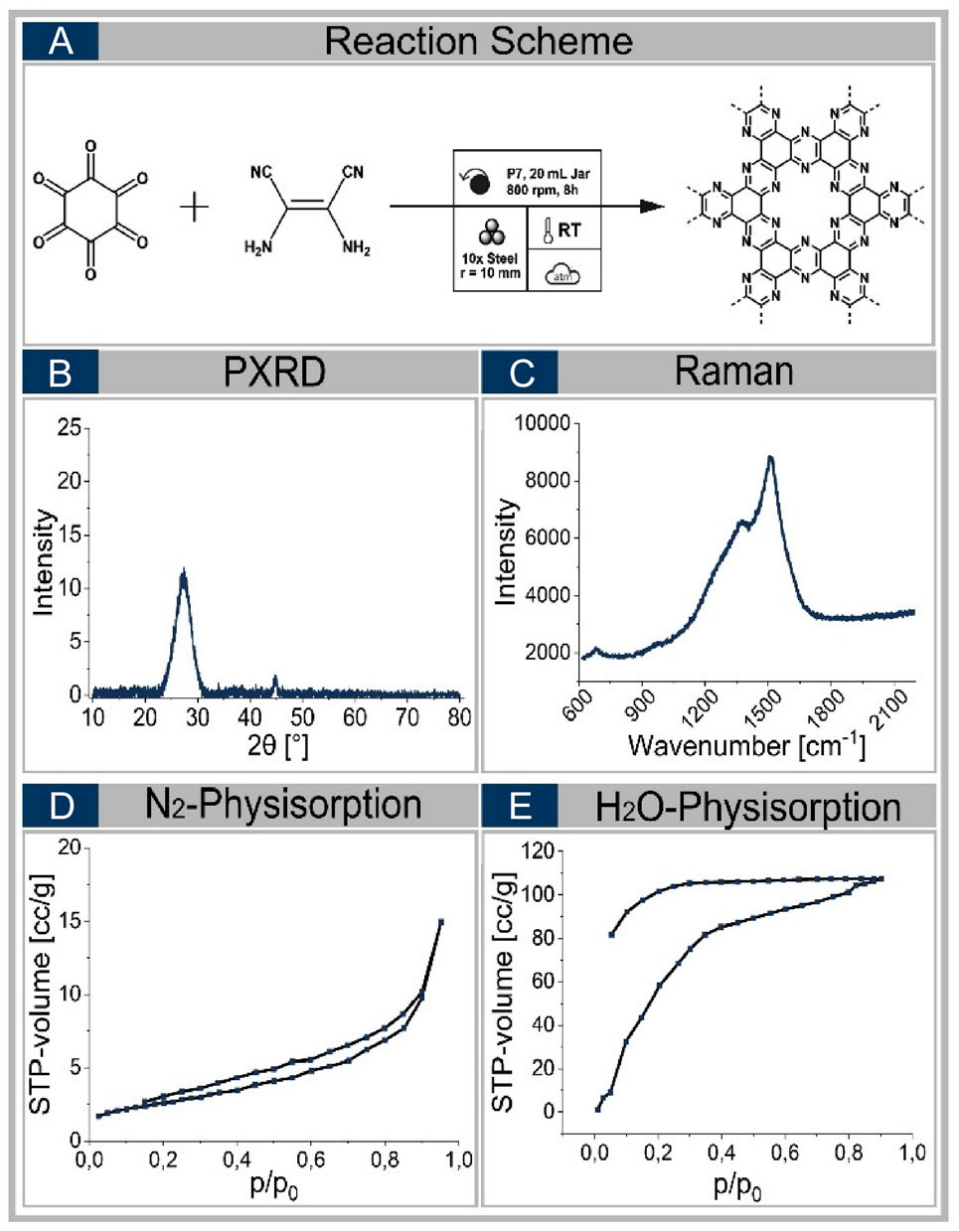
Analysis results of the two‐compound one‐pot strategy in the P7 ball mill (A). It contains the X‐ray diffractogram (B), the Raman spectrum (C), the nitrogen physisorption isotherm (D), and the water physisorption isotherm (E).

According to the Raman spectrum, the C_2_N material has formed successfully, with the shifted D‐ and G‐signals observable at the same positions as in the HAT‐CN derived material. The X‐ray diffractogram is comparable to it as well, revealing an amorphous C_2_N material, with one broad signal at 2*θ* = 27°. The surface parameters are comparable to the material derived from HAT‐CN (see Figure 5). We concluded that the material, obtained by the two‐compounds conversion, is equal to the material, obtained by the HAT‐CN conversion.

SEM/EDX spectroscopy, elemental analysis and XPS confirm a similar composition with >30 wt% of nitrogen content. This two‐step one‐pot conversion provides a viable shortcut to C_2_N synthesis, reducing synthetic steps and saving energy, waste and cost, while providing a material of equal quality.

### 2.3 Solid‐State NMR Analysis

**FIGURE 6:**
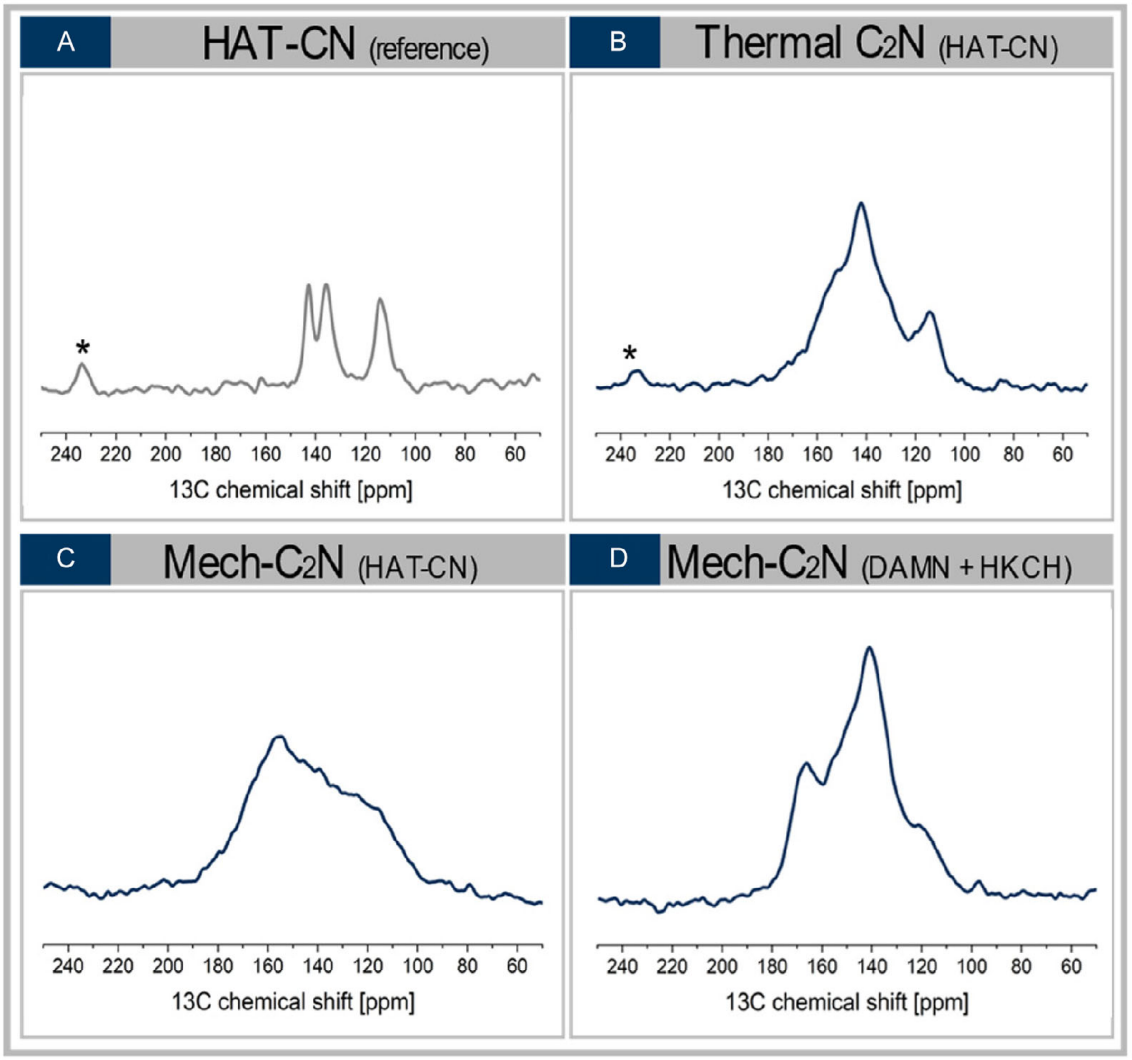
^13^C solid‐state NMR spectra of HAT‐CN (A), C_2_N materials prepared from HAT‐CN using a thermal (B) and mechanochemical (C) approach and the C_2_N material obtained from the two‐compound one‐pot strategy (D). Asterisks (*) indicate spinning sidebands from MAS at 9 kHz.

Solid‐state ^13^C NMR spectroscopy was employed to compare the structural characteristics of materials obtained via thermal conversion, mechanochemical conversion, and the two‐compound one‐pot synthesis route (Figure 6B‐D). For reference, the spectra of HAT‐CN as well as those of the individual one‐pot precursors are shown in Figure 6A and the ESI Figure S7, respectively.

All product spectra show broad resonances between 100 and 180 ppm, characteristic of aromatic and heteroaromatic carbon environments and indicative of disordered carbon networks. In contrast, pristine HAT‐CN (Figure 6A) shows well‐defined resonances in agreement with literature data [42],reflecting its molecular nature prior to conversion. Mechanochemically converted HAT‐CN (Figure 6C) displays a single broad resonance at ˜ 156 ppm, characteristic of extended C_2_N‐type networks [43, 44]. The absence of additional resolved signals at lower chemical shifts indicates a more complete transformation than in the thermally treated sample (Figure 6B), which retains residual HAT‐CN, as indicated by the signal at 114 ppm [42]. The dominant resonance at ˜146 ppm is attributed to the inner bulk structure of HAT‐CN forming the main framework of the C_2_N material [42]. By contrast, the mechanochemical two‐compound one‐pot product (Figure 6D) exhibits multiple distinct features, indicating increased chemical heterogeneity. In addition to C_2_N‐like environments, resonances at ˜166 ppm suggest side‐product functionalities such as carboxylic acids or esters formed by incorporation of water released during condensation, while a signal at 97 ppm is assigned to unreacted hexaketocyclohexane octahydrate [45, 46]. Overall, the obtained ^13^C solid‐state NMR spectral pattern (Figure 6B‐D) underlines the formation of C_2_N‐type materials for all synthesis routes, while revealing pronounced differences in structural homogeneity between direct, thermal, and condensation‐based approaches.

### 2.4 Green Metrics Calculation

To demonstrate the sustainability of the new mechanochemical pathway, a green metrics calculation was performed (see Table 1).

**TABLE 1:** Quantitative green metrics calculation, based on the synthesis of 1000 g of C_2_N material.

Reaction type	Yield	PMI	MP	Energy demand	Global warming potential
	[%]			[MWh]	**[kgCO**_**2**_ **‐eq]** b **^,^** c
*HATCN → C*_2_ *N* Classical^a^	50.0	2.0	50.0	34.2	6532.2
Mechanochemical	86.2	379.2	0.3	2.8	619.1
*DAMN + HKCH → C*_2_ *N* Classical^a^	25.0	638.7	0.2	36.2	9842.2
Mechanochemical	94.6	291.9	0.3	2.8	890.1

a This values were calculated, based on Walczak et al.'s procedure at 550°C [25].

b $\mathrm{PMI}=\frac{\text{Total}\mathrm{mass}\;\mathrm{used}\;\mathrm{in}\text{process}}{\mathrm{mass}\;\mathrm{of}\text{prodct}}$,

c $\mathrm{MP}=\frac{1}{\mathrm{PMI}}\cdot 100$.

From these calculations, we conclude that the mechanochemical routes to C_2_N‐type materials are considerably greener, primarily due to its reduced energy demand and no solvent use for synthesis. In the classical approach, most of the energy consumption arises from the high‐temperature pyrolysis step, which additionally requires an inert atmosphere. The major solvent contribution stems from the wet‐chemical synthesis of HAT‐CN [25]. In contrast, the mechanochemical method just uses HCl and water for the work‐up. Furthermore, the need for solvent‐intensive precursor synthesis is eliminated.

## 3 Conclusion

We presented a new near‐ambient strategy to convert HAT‐CN into a C_2_N‐type material, based on mechanochemistry instead of inert pyrolysis. The optimal conditions turned out to be in the P7 planetary ball mill at 800 rpm for 3 h, using steel as milling material. According to the results from the variation of mechanochemical parameters, the reaction pathway is highly sensitive to the mechanical energy impact, as the differences in yield were significant by its variation.

Additionally, we were able to develop a ball milling strategy to convert the precursors hexaketocyclohexane and diaminomaleonitrile directly into the C_2_N‐type material, through increasing the total energy input by extending the milling time. This reaction pathway allows effectively to skip the formation of HAT‐CN as intermediate for further conversion. Finally, we showed that the mechanochemical synthetic pathway is significantly greener than the classical strategies, mainly due to the reduced consumption of energy.

## 4 Experimental Section

### 4.1 General Information

The precursor chemicals 1,4,5,8,9,11‐hexaazatriphenylene‐hexacarbonitrile (HAT‐CN), respectively hexaketocyclohexane‐octahydrate and diaminomaleonitrile were obtained from commercial suppliers and used without further purification. Acetone for the work‐up and hydrochloric acid for the removal of experiment‐specific iron abrasions were obtained commercially. Water was used in its deionized form. The mechanochemical synthesis step was carried osut in a Retsch MM500 mixer ball mill, a Fritsch Pulverisette 7 premium line planetary ball mill or a Retsch high energy Emax ball mill. The milling balls’ material was varied in the different experiments, including polypropylene (PP), silicium nitride (Si_3_N_4_), zirconium dioxide (ZrO_2_), steel and tungsten carbide (WC). The vessel materials were adjusted with respect to the milling balls’ material, e.g., steel vessels for steel balls and tungsten carbide vessels for tungsten carbide balls. Zircon vessels were used for balls, made of zircon, silicium nitride, and polypropylene.

### 4.2 Synthesis Procedure in the MM500 Mixer Ball Mill

600 mg of 1,4,5,8,9,11‐hexaazatriphenylene‐hexacarbonitrile was placed into a 25 mL vessel with two milling balls (*Ø* = 10 mm) of the respective material (see General Information). The mixture was milled in a MM500 mixer ball mill at 35 Hz for 180 min. The resulting black powder was worked up by filtration and washed with water and acetone, until the filtrate became colorless. For experiments with steel milling balls, an additional washing step with 50 mL 18% hydrochloric acid was included, to remove potential iron abrasion and subsequently washed with 300 mL water. After washing, the samples were dried at 80°C overnight and characterized. The milling parameters, time, frequency, and material were varied (see table S1).

### 4.3 Synthesis Procedure in the P7 Planetary Ball Mill

600 mg of 1,4,5,8,9,11‐hexaazatriphenylene‐hexacarbonitrile was placed into a 20 mL vessel with ten milling balls (*Ø* = 10 mm) of the respective material (see General Information). The mixture was milled in a P7 planetary ball mill at 800 rpm for 180 min. The resulting black powder was worked up by filtration and washing with water and acetone, until the filtrate became colorless. For experiments with steel milling balls, an additional washing step with 50 mL of 18% hydrochloric acid was included, to remove potential iron abrasion and subsequently washed with 300 mL water. After washing, the samples were dried at 80°C overnight and characterized. The milling parameters, time, frequency and material were varied (see Table 1). For the LAG‐template assisted synthesis, 600 mg of potassium chloride was added, with 0.15 mL (*µ* = 0.25) of the respective solvent.

### 4.4 Synthesis Procedure in the Emax High Energy Ball Mill

800 mg of 1,4,5,8,9,11‐hexaazatriphenylene‐hexacarbonitrile was placed into a 50 mL vessel with ten milling balls (*Ø* = 10 mm) of the respective material (see General Information). The mixture was milled in a Retsch high‐energy Emax ball mill at 1200 rpm for 180 min. The resulting black powder was worked up by filtration and washing with water and acetone, until the filtrate became colorless. For experiments with steel milling balls, an additional washing step with 50 mL of 18% hydrochloric acid was included, to remove potential iron abrasion and subsequently washed with 300 mL water. After washing, the samples were dried at 80°C overnight and characterized.

### 4.5 Synthesis Procedure for the Two‐Step One‐Pot Transformation

400 mg of hexaketocyclohexane and 1.1 g of diaminomaleonitrile were placed in a 20 mL steel milling jar, with ten steel milling balls (*Ø* = 10 mm) and milled at 800 rpm in a P7 planetary ball mill for 8 h. The resulting black powder was worked up by stirring in 50 mL concentrated hydrochloric acid overnight and subsequent washing with 300 mL of water. It was dried at 80°C overnight and characterized.

### 4.6 Characterization Methods

The dried samples were characterized via Raman spectroscopy, X‐ray photoelectron spectroscopy (XPS), powder X‐ray diffraction (PXRD), physisorption and vaporsorption measurements, and thermogravimetric analysis (TGA). Raman spectroscopy was carried out with a Renishaw inVia Qontor microscope through the 50x objective (NA = 0.50, 8.2 mm free working distance) with an exposure time of 1 s. 50 acquisitions at 100% signal amplification and 100% aperture opening were used with an excitation wavelength of 532 nm at 10% intensity. The respective center wavelength was set to 1500 cm^−1^ with a 1800 l/mm grating. For the XRD‐measurement, a PANalytical Xpert Pro device with a Cu‐K_α_1 source (*λ* = 1.5418 Å) and increments of 0.0201° was used for a range of 2*θ* = 10°‐80°. To characterize the porosity, a Quantachrome's Quadrasorb evo surface area and pore size analyzer was used with nitrogen at 77 K and water at 298 K as adsorptives. Prior to the measurement, 50–100 mg of the respective sample was degassed in a 9 mm bulbless cell under vacuum at 423 K for at least 24 h. The adsorption branches were measured with 26 and the desorbtion branch with 17 points, respectively. A Nexsa G2 surface analysis system was used for the X‐ray photoelectron spectra (XPS). As source, a monochromated, micro‐focused, high efficiency A1 K X‐ray source was used. A 180°, double‐focus, hemispherical detector with 128 channels served as analyzer. The spectra were recorded by scanning 20 times with keV. A JEOL JSM IT800SHL scanning electron microscope was used for recording the SEM images as well as the energy‐dispersive X‐ray spectra (EDX). It contains a secondary electron detector at 5 kV. An Oxford Ultim Max with a silicon drift detector was used to record the EDX spectra at a working distance of 10 mm.

All solid‐state NMR spectra except for diaminomaleonitrile were acquired on a 300 MHz Bruker Avance III HD spectrometer with a 4 mm H/C double resonance probe under magic angle spinning (MAS). A zgbs pulse sequence employing background suppression and proton decoupling during acquisition was utilized to record ^13^C MAS spectra at 9 kHz spinning. Spectra were measured with 20 s relaxation delay, 4096 scans and an acquisition time of 34.4 ms. 90°‐Pulses for ^13^C were adjusted to 4 µs. Proton decoupling was applied using the tppm15 [47] decoupling scheme. The spectrum of diaminomaleonitrile was acquired on a 400 MHz Bruker Avance III DNP spectrometer with a 3.2 mm ^1^H/X/Y triple resonance low temperature probe at nominally 293 K. Spectra were recorded at 12 kHz spinning. For the ^1^H→^13^C CP MAS spectra, a 90° excitation pulse of 2.5 µs was applied on the ^1^H channel. The contact time was set to 2 ms. During the contact, a linear 100‐50% ramp was applied on the ^1^H channel. The recycle delay was set to 3 s. Spectra were recorded with 256 scans employing an acquisition time of 30 ms during which heteronuclear spinal64 [48] decoupling was applied.

Spectra were acquired and processed using the Bruker TopSpin software and further processed using Mestrelab Research MestReNova 14.2.1. A line broadening of 200 Hz was applied to all spectra.

## Supporting Information

Additional supporting information can be found online in the Supporting Information section.

## Funding

This study was supported by the Bundesministerium für Bildung und Forschung (grant 03SF0737B).

## Conflicts of Interest

The authors declare no conflicts of interest.

## Supporting information

Supplementary Material

## Data Availability

The data that supports the findings of this study are available in the supplementary material of this article.
