# Supplementary material for: Mechanochemical Near‐Ambient Synthesis of C2N Materials From HAT‐CN and its Precursors
Source: ChemSusChem. 2026 May 10;19(9):e70678. doi: 10.1002/cssc.70678 (PMC13158639; doi:10.1002/cssc.70678)
Supplement: Supplementary file 1 — Supplementary Material [file CSSC-19-e70678-s001.pdf]

SUPPORTING INFORMATION

Mechanochemical near-ambient synthesis of C_2N materials from HAT-CN and its precursors

Pascal Dippner[a], Sven Grätz[a], Jonas Lins[b], Torsten Gutmann[b,c] and Lars Borchardt[a]*

[a] [a] P. Dippner, S. Grätz, and L. Borchardt
Inorganic chemistry I
Ruhr-Universität Bochum
Universitätsstraße 150, 44801 Bochum, Germany
E-mail: lars.borchardt@ruhr-uni-bochum.de
[b] J. Lins and T. Gutmann
Eduard-Zintl-Institute for Inorganic and Physical Chemistry
Technische Universität Darmstadt
Peter-Grünberg-Straße 8, 64287 Darmstadt, Germany
[c] T. Gutmann
Department of Chemistry, Physical Chemistry
Universität Paderborn
Warburger Straße 100, 33098 Paderborn

Table of contents

1. Synthetic procedures 3
1.1 General information 3
1.2 MM500 Mixer ball mill 4
1.3 P7 planetary ball mill 4
1.4 Emax high-energy ball mill 4
1.5 Two-compound one-pot conversion 5
1.6 Characterization methods 5
2. Screening of mechanochemical parameters 6
3. Analysis results 7
3.1. Characterization of the product 7
3.1.1. HAT-CN conversion 7
3.1.2. 2-step one-pot synthesis 8
3.2. Compositions and surface parameters 9
3.3. Energetic considerations 10
3.4. Solid-state NMR 11

1. Synthetic procedures

1.1 General information

The precursor chemicals 1,4,5,8,9,11-Hexaazatriphenylene-hexacarbonitrile (HAT-CN), hexaketocyclohexane-octahydrate and diaminomaleonitrile were obtained from commercial suppliers and used without further purification. Acetone for the work-up and hydrochloric acid for the removal of experiment-specific iron abrasions were obtained commercially. Water was used in its deionized form. The mechanochemical synthesis step was carried out in a Retsch MM500 mixer ball mill, a Fritsch Pulverisette 7 premium line planetary ball mill or a Retsch high energy Emax ball mill (Fig. S1). The milling balls' material was varied in the different experiments, including polypropylene (PP), silicium nitride (Si_3N_4), zirconium dioxide (ZrO_2), steel and tungsten carbide. The vessel materials were adjusted with respect to the milling balls' material, e.g. steel vessels for steel balls and tungsten carbide vessels for tungsten carbide balls. Zircon vessels were used for balls, made of zircon, silicium nitride and polypropylene.

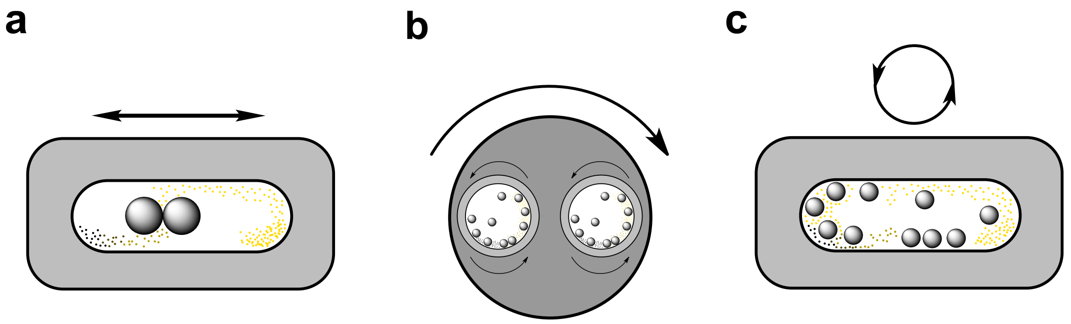

Figure S1. Schematic representation of used ball mall types for the investigation of mechanochemical parameters, from left to right: MM500 mixer ball mill, P7 planetary ball mill, Emax high energy ball mill.[1,2]

1.2 MM500 Mixer ball mill

400 mg of 1,4,5,8,9,11-Hexaazatriphenylene-hexacarbonitrile was placed into a 25 mL vessel with two milling balls (Ø = 10 mm) of the respective material (see General Information). The mixture was milled in a MM500 mixer ball mill at 35 Hz for 180 minutes. The resulting black powder was worked up by filtration and washed with water and acetone, until the filtrate became colorless. For experiments with steel milling balls, an additional washing step with 50 mL of hydrochloric acid was included, to remove potential iron abrasion and subsequently washed with 300 mL water. After washing, the samples were dried at 80 °C overnight and characterized. The milling parameters, time, frequency and material were varied (see table S1).

1.3 P7 planetary ball mill

600 mg of 1,4,5,8,9,11-Hexaazatriphenylene-hexacarbonitrile was placed into a 20 mL vessel with ten milling balls (Ø = 10 mm) of the respective material (see General Information). The mixture was milled in a P7 planetary ball mill at 800 rpm for 180 minutes. The resulting black powder was worked up by filtration and washing with water and acetone, until the filtrate became colorless. For experiments with steel milling balls, an additional washing step with 50 mL of hydrochloric acid was included, to remove potential iron abrasion and subsequently washed with 300 mL water. After washing, the samples were dried at 80 °C overnight and characterized. The milling parameters, time, frequency and material were varied (see table 1). For the LAG-template assisted synthesis, 600 mg of potassium chloride was added, with 0.15 mL (η = 0.25 µL/mg) of the respective solvent.

1.4 Emax high-energy ball mill

800 mg of 1,4,5,8,9,11-Hexaazatriphenylene-hexacarbonitrile was placed into a 50 mL vessel with ten milling balls (Ø = 10 mm) of the respective material (see General Information). The mixture was milled in a Retsch high-energy Emax ball mill at 1600 rpm for 180 minutes. The resulting black powder was worked up by filtration and washing with water and acetone, until the filtrate became colorless. For experiments with steel milling balls, an additional washing step with 50 mL of hydrochloric acid was included, to remove potential iron abrasion and subsequently washed with 300 mL water.

1.5 Two-compound one-pot conversion

400 mg of hexaketocyclohexane and 1.1 g of diaminomaleonitrile were placed in a 20 mL steel milling jar, with ten steel milling balls (Ø = 10 mm) and milled at 800 rpm in the P7 planetary ball mill for eight hours. The resulting black powder was worked up by washing it with water and hydrochloric acid. It was dried at 80 °C overnight and characterized.

1.6 Characterization methods

Raman spectroscopy was carried out with a Renishaw inVia Qontor microscope through the 50x objective (NA = 0.50, 8.2 mm free working distance) with an exposure time of 1 s. 50 acquisitions at 100% signal amplification and 100% aperture opening were used with an excitation wavelength of 532 nm at 10% intensity. The respective center wavelength was set to 1500 cm-1 with a 1800 l/mm grating. For the XRD-measurement, a PANalytical Xpert Pro device with a Cu-Kα1 source (λ = 1.5418 Å) and increments of 0.0201° was used for a range of 2θ = 10-80°. To characterize the porosity, a Quantachrome's Quadrasorb evo surface area and pore size analyzer was used with nitrogen at 77 K and water at 298 K as adsorptives. Prior to the measurement, 50-100 mg of the respective sample was degassed in a 9 mm bulbless cell under vacuum at 423 K for at least 24 hours. The adsorption branches were measured with 26 and the desorbtion branch with 17 points, respectively. A Nexsa G2 surface analysis system was used for the X-ray photoelectron spectra (XPS). As source, a monochromated, micro-focused, high efficiency A1 K X-ray source was used. A 180 °, double-focus, hemispherical detector with 128 channels served as analyzer. The spectra were recorded by scanning 20 times with keV. A JEOL JSM IT800SHL scanning electron microscope was used for recording the SEM images as well as the energy-dispersive X-ray spectra (EDX). It contains a secondary electron detector at 5 kV. An Oxford Ultim Max with a silicon drift detector was used to record the EDX spectra at a working distance of 10 mm. All solid-state NMR spectra except for diaminomaleonitrile were acquired on a 300 MHz Bruker Avance III HD spectrometer in a 4 mm broad band probe under 9 kHz magic angle spinning. A zgbs experiment with background suppression and proton decoupling during acquisition was utilized. Spectra were recorded with 20 s relaxation delay, 4096 scans and an acquisition time of 34.4 ms. 90°-Pulses for 13C were 4 µs long at 200 W power, for proton decoupling using the tppm15_13[3] scheme pulses were 7 µs at 55 W.

2. Screening of mechanochemical parameters

Table S1. Yields and parameters for screening experiments with various mills and mechanochemical parameters. I represents the conversion of HAT-CN, II represents the conversion of the HAT-CN precursors, V represents HAT-CN conversion experiments with a cumulative energy of E_{cum} = 800 kJ.

Material/LAG	Material	Mill	Time [min]	Frequency [rpm]	Yield [wt%]
I-1	PP	MM500	180	35[b]	0
I-2	Si_3N_4	MM500	180	35[b]	3
I-3	ZrO_2	MM500	180	35[b]	13
I-4	Steel	MM500	180	35[b]	11
I-5	PP	P7	180	800	0
I-6	Si_3N_4	P7	180	800	31
I-7	ZrO_2	P7	180	800	81
I-8	Steel	P7	180	400	0
I-9	Steel	P7	180	600	1
I-10	Steel	P7	30	800	0
I-11	Steel	P7	60	800	31
I-12	Steel	P7	120	800	80
I-13	Steel	P7	180	800	86
I-14	ZrO_2	Emax	180	800	0
I-15	ZrO_2	Emax	180	1200	11
I-16	ZrO_2	Emax	180	1600	85
I-17	Steel	Emax	180	1200	37
I-18	WC	Emax	180	800	16[a]
I-19	WC	Emax	180	1200	109[a]
II-1	ZrO_2	P7	640	800	87
II-2	Steel	P7	640	800	95
V-1	PP	P7	9942	400	0
V-2	Si_3N_4	P7	2396	400	0
V-3	ZrO_2	P7	1339	400	0
V-4	Si_3N_4	P7	710	600	0
V-5	Steel	P7	968	400	0
V-6	Si_3N_4	P7	300	800	0
V-7	ZrO_2	P7	300	600	0
V-8	Steel	P7	287	600	16
V-9	ZrO_2	P7	167	800	85
V-10	Steel	P7	121	800	91
V-11	Steel	P7	85	900	91
V-12	Steel	P7	62	1000	92

[a] The yield corresponds to the sum of the mass of carbon material and the tungsten abrasion.
[b] The unit for this frequency is Hz, as the used mill is a MM500 mixer ball mill.

3. Analysis results

3.1. Characterization of the product

3.1.1. HAT-CN conversion

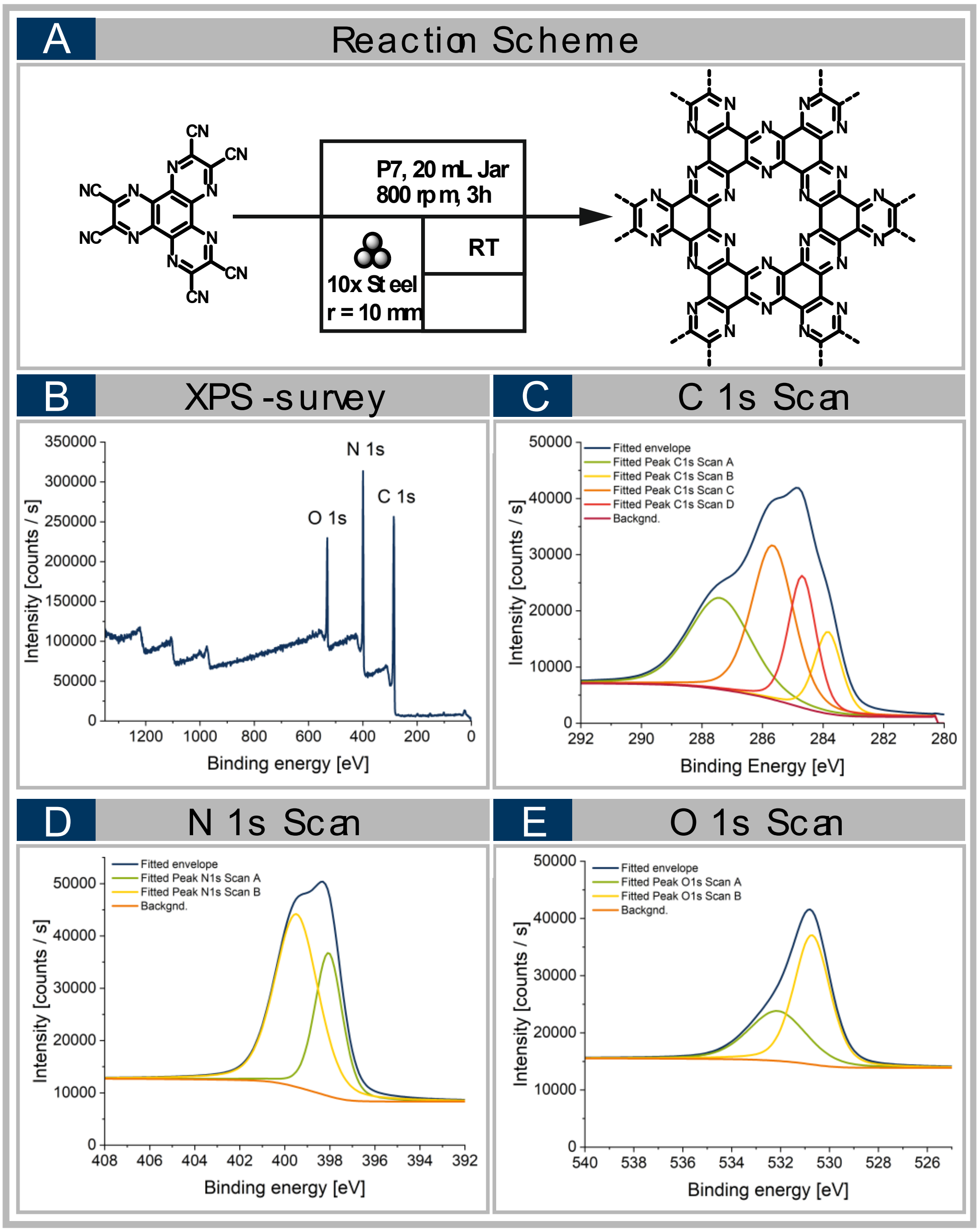

Figure S2. Reaction scheme of the HAT-CN conversion and the respective XPS-spectra of the product.

3.1.2. 2-step one-pot synthesis

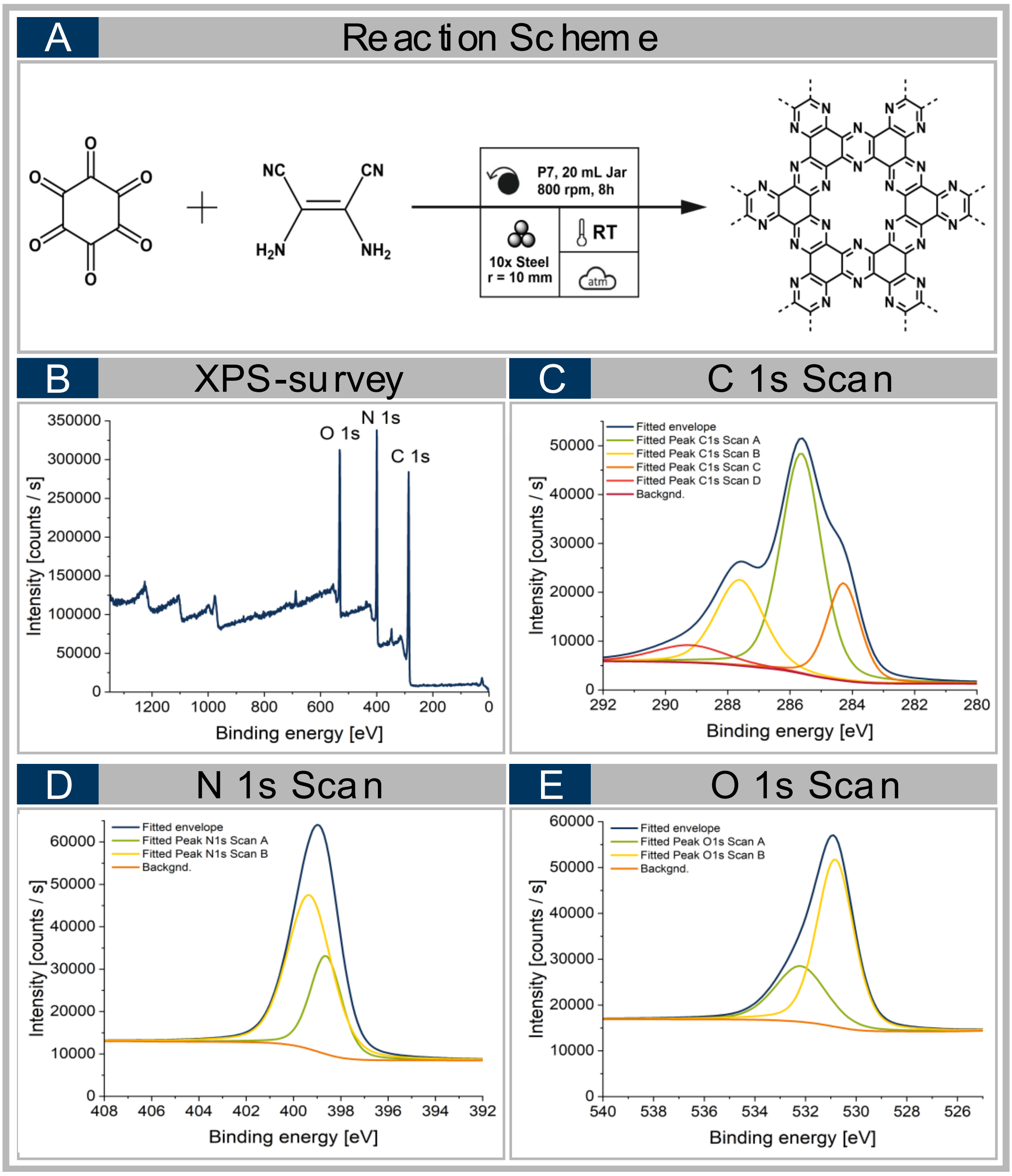

Figure S3. Reaction scheme of the two-compound one-pot conversion and the respective XPS-spectra of the product.

3.2. Compositions and surface parameters

Table S2. Analysis results for the surface parameters and elemental compositions of screening experiments with various mills and mechanochemical parameters. The surface parameters were determined by Nitrogen physisorption measurement.

Entry	SSA [m^2/g]	Pore Volume [cc/g]	Elemental analysis Method	C [wt%]	N [wt%]	O [wt%]
**I-3**	13.58	0.04	SEM/EDX	53.7	38.6	6.21
			XPS	53.9	27.4	14.2
			EA	47.5	36.1	-
**I-4**	5.17	0.03	SEM/EDX	51.5	35.1	8.7
**I-6**	5.47	0.01	SEM/EDX	54.0	37.9	7.5
**I-7**	21.02	0.04	SEM/EDX	53.8	36.9	6.2
			XPS	54.8	30.9	10.9
			EA	45.3	34.4	-
**I-11**	1.86	0	SEM/EDX	47.9	41.3	10.1
**I-12**	12.22	0.2	SEM/EDX	51.7	38.7	9.4
**I-13**	17.06	0.03	SEM/EDX	51.0	40.7	7.9
			XPS	53.1	33.5	13.5
			EA	47.8	34.0	-
**I-15**	19.76	0.04	SEM/EDX	54.2	40.1	5.5
**I-16**	23.51	0.04	SEM/EDX	52.31	39.95	7.34
			XPS	57.9	31.0	11.1
			EA	46.2	35.9	-
**I-17**	-	-	SEM/EDX	51.3	36.6	10.9
**I-19**[a]	37.34	0.05	SEM/EDX	25.3	5.2	4.9
**II-1**	1.14	0	SEM/EDX	44.4	37.1	10.5
**II-2**	-	-	SEM/EDX	44.4	34.7	20.9
			XPS	50.8	29.6	17.8
			EA	38.7	31.3	-

[a] The sample contains 56.11 wt % of W, caused by abrasion from intense milling.

3.3. Energetic considerations

A minimum amount of energy input is required to induce the transformation of HAT-CN. A good indicator for the success of the reaction is the color of the product, which has a brownish tone, if the energy input was insufficient, for example in the MM-500 with polypropylene balls. ^{13}C solid state NMR confirmed the incomplete reaction of the intermediate product, which resembles a slightly broadened spectrum of the pure HAT-CN. In contrast the end product shows significant broadening and a signal with higher chemical shifts.

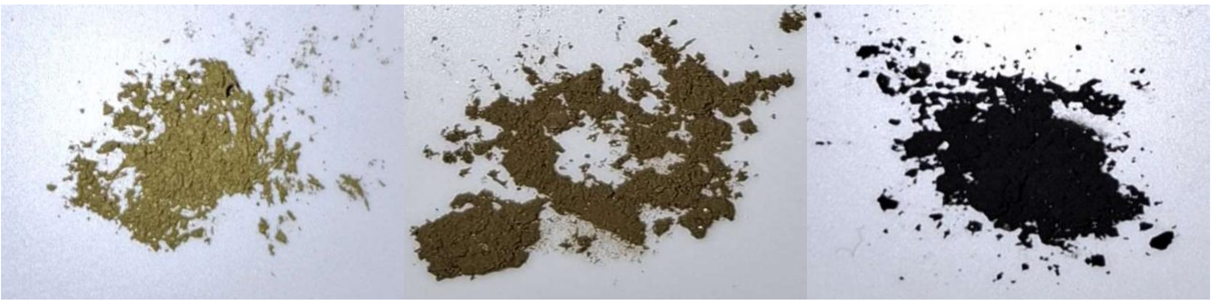

Figure S5. Pure HAT-CN with a yellow color (left), after ball-milling treatment with insufficient energy input (middle) and after ball milling treatment with sufficient energy input (right).

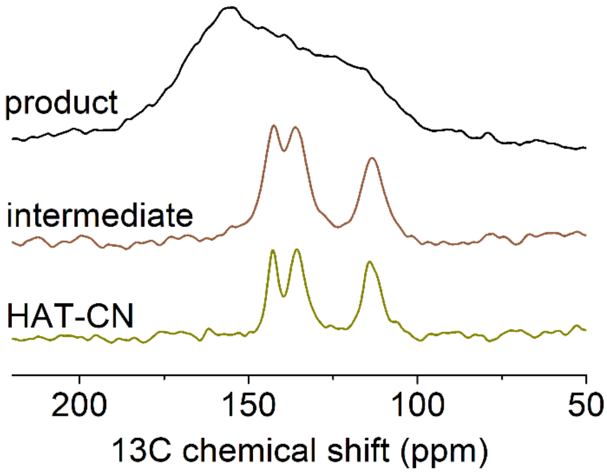

Figure S6. ^{13}C solid-state NMR spectra of pure HAT-CN with a yellow color (bottom), the intermediate after ball-milling treatment with insufficient energy input in brown (middle) and the end product after ball milling treatment with sufficient energy input in black (top).

3.4. Solid-state NMR

For comparison with the products, the educts HAT-CN (Fig. 5 D) as well as hexaketocyclohexane and diaminomaleonitrile (Fig. S7) were measured using ^{13}C solid-state-NMR, using the zgbs pulse sequence. Diaminomaleonitrile (Fig. S7, blue/bottom) was measured using ^{13}C CP MAS NMR and features three distinct signals at 119, 111 and 105 ppm.

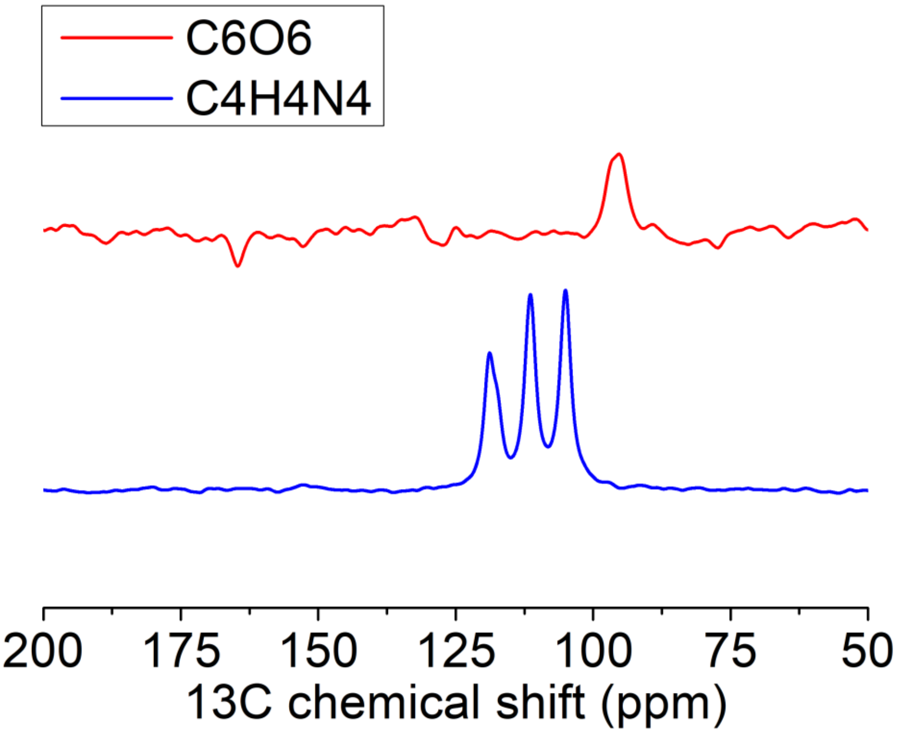

Figure S7. ^{13}C solid-state NMR spectra of the two educts hexaketocyclohexane (red, top) and diaminomaleonitrile (blue, bottom).

Hexaketocyclohexane was found to show a peak around 96 ppm (Fig. S7, red/top). While this shift is unusual for a ketone, it was found that commercially available hexaketocyclohexane octahydrate structurally represents dodecahydroxycyclohexane, which would show an expected shift in that range.[4,5]

All product spectra show broad signals in the chemical shift range between 100 ppm and 180 ppm, typical for aromatic and heteroaromatic systems. The intense signal broadening indicates highly disordered structures. In contrast, the educt HAT-CN shows three distinct signals at 114, 136 and 143 ppm (Fig. 5 D), in agreement with the literature[6].

The spectrum of the thermal conversion product (Fig. 5 B) has a major peak around 142 ppm with a right shoulder around 130 ppm and a smaller peak around 114 ppm which probably corresponds to non-reacted HAT-CN. A shoulder on the left around 152 ppm would correspond to the bulk of the C_2N material.

In the mechanochemically prepared product spectrum (Fig. 5 C) there is only one broad and featureless signal with an asymmetric peak around 156 ppm that would also correspond to the bulk of the C_2N material. The absence of other defined signals of lower chemical shift indicates a more complete reaction in this sample. In the spectrum for the mechanochemically prepared one-pot product there are three major features visible: A maximum peak at 141 ppm with a left shoulder between 150 to 155 ppm, a signal at 166 ppm, and a smaller right shoulder around 121 ppm. The small shoulder around 121 ppm together with the maximum at 141 ppm could correspond to HAT-CN that has formed as an intermediate product, while the broad shoulder around 155-150 ppm fits to the signals of the C_2N material observed in the other two approaches. An additional signal around 166 ppm suggests side-product functionalities such as carboxylic acids, esters or amides formed from the two educts hexaketocyclohexane and diaminomaleonitrile, by incorporation of water released during the condensation. The small signal around 97 ppm most likely refers to unreacted hexaketocyclohexane.

[1] O. F. Jafter, S. Lee, J. Park, C. Cabanetos, D. Lungerich, *Angewandte Chemie (International ed. in English)* **2024**, *63*, e202409731.

[2] J. Rios, A. Restrepo, A. Zuleta, F. Bolívar, J. Castaño, E. Correa, F. Echeverria, *Metals* **2021**, *11*, 1621.

[3] A. E. Bennett, C. M. Rienstra, M. Auger, K. V. Lakshmi, R. G. Griffin, *The Journal of Chemical Physics* **1995**, *103*, 6951.

[4] G. K. Lim, K. Fujii, K. D. M. Harris, D. C. Apperley, *Crystal Growth & Design* **2011**, *11*, 5192.

[5] Y. Lu, X. Hou, L. Miao, L. Li, R. Shi, L. Liu, J. Chen, *Angewandte Chemie* **2019**, *131*, 7094.

[6] W. Pickhardt, M. Wohlgemuth, S. Grätz, L. Borchardt, *The Journal of organic chemistry* **2021**, *86*, 14011.
